# Docosahexaenoic acid and the preterm infant

**DOI:** 10.1186/s40748-017-0061-1

**Published:** 2017-12-12

**Authors:** Stephanie L. Smith, Christopher A. Rouse

**Affiliations:** 1 Uniformed Services University of Health Sciences, 4301 Jones Bridge, Bethesda, MD 20814 USA; 2 Walter Reed National Military Medical Center, 8901 Rockville Pike, Bethesda, MD 20814 USA

**Keywords:** Docosahexaenoic acid, Preterm infants, DHA supplementation, Breast milk, Inflammation, Neurodevelopment

## Abstract

Docosahexaenoic acid (DHA) is a long chain poly-unsaturated fatty acid (LCPUFA) that has a role in the cognitive and visual development, as well as in the immune function of newborns. Premature infants are typically deficient in DHA for several reasons, to include fetal accretion of DHA that typically occurs during the third trimester. These premature infants are reliant on enteral sources of DHA, most commonly through breast milk. The DHA content in breast milk varies in direct correlation with maternal DHA intake and mothers consuming a Western diet typically have lower levels of DHA in their breast milk. Maternal DHA supplementation and direct supplementation of DHA to the infant has been tried successfully but there are still conflicting results on the optimal dosage and method of delivery of DHA to the infant. This has led to inconsistent results in trials evaluating the effects of DHA supplementation to the preterm infant in terms of cognitive and immunological outcomes. While short-term benefits have been seen in several studies, long-term benefits are not consistent. Future studies continue to be needed to optimize DHA intake in our premature infants.

## Background

Docosahexaenoic acid (DHA) is a long chain poly-unsaturated fatty acid (LCPUFA) that has a role in the cognitive and visual development, as well as in the immune function of newborns [1]. DHA is derived from α-linolenic acid (ALA), an essential omega-3 fatty acid, through the enzyme **∆6-desaturase** [1]. ALA, along with its omega-6 counterpart linoleic acid (LA), are essential fatty acids that must be obtained from the diet as the human body is unable to synthesize them. CLA is the most common essential fatty acid consumed in the Western diet and can be found in vegetable oils, nuts and seeds. ALA is commonly found in flaxseed, walnuts and soy while DHA is found in fatty fishes such as salmon and tuna. While there is no consensus on the recommended DHA intake for pregnant or lactating women, several organizations, including the March of Dimes, American Academy of Pediatrics, and the Food and Agriculture Organization of the United Nations agree that pregnant and lactating women should have a minimal intake of 200–300 mg of DHA per day [2]. However, it is uncommon for women eating a Western diet to consume the recommended amount of DHA. A study published in 2017 by Nordgren et al. focused on evaluating the omega-3 fatty acid intake in pregnant women as well as women of childbearing age in the United States [3]. The authors found that DHA intake in both pregnant and non-pregnant women was well below the recommended minimal intake of 200–300 mg/day [3]. On average, a pregnant women’s DHA intake in the United States was only 66 mg/day and only 9.0% of women took DHA supplements during pregnancy [3]. Lower levels of DHA intake were correlated with lower economic status and education [3]. In addition, DHA crosses the placenta with the highest fetal accretion rates of LA, AA and DHA occur in the last 5 weeks of pregnancy [4]. Fetuses born to women who consume a typical Western diet have a DHA accretion rate of approximately 42–67 mg/day in the last trimester, though this rate may not be ideal based on the low DHA intake and high LA intake of the Western diet [4, 5].

Following delivery, infants rely on enteral sources to meet their DHA requirement, whether through breast milk or formula. The DHA content of breast milk is correlated to maternal intake of DHA. To add to this, obesity is becoming more prevalent in the United States and has an effect on the omega-3 fatty acids in breast milk [6]. Breast milk analyzed from obese mothers with BMI >30 kg/m^2^, was significantly lower in long-chain n-3 FA and had a higher n-6:n-3 ratio. These findings were also seen in the plasma of breastfed neonates of obese mothers [6]. The n-3 concentration, which includes the concentration of DHA, in the breast milk of obese mothers could be improved by enrolling the mothers into a clinic for weight control and dietary support [6]. Vegetarian and vegan diets are also becoming more prevalent in today’s society and it is also crucial to consider these diets’ effects on DHA. Sanders reviewed published studies on the DHA status in vegetarians and vegans and found a lower DHA status in vegetarians and vegans compared to omnivores [7]. The concentration of DHA in breast milk is important because of DHA’s contribution to brain and visual development as well as its affect on the immune system, which is vital for the premature infant. The objective of this paper is to review DHA and the premature infant looking specifically at: 1) DHA supplementation and its effect on breast milk; 2) DHA and the premature infant; 3) DHA and its effect on the neurodevelopment in the premature infant; and 4) DHA and its effect on the immune response in the premature infant.

### DHA and Breast milk

Following delivery, infants become reliant on an enteral source of DHA, commonly through maternal breast milk. DHA content in breast milk is affected by several factors including maternal nutrition, gestational age at delivery, genetics, and the stage of lactation. The DHA content in breast milk varies in direct correlation with maternal DHA intake. The worldwide mean DHA concentration of breast milk is 0.32 ± 0.22% (mean ± standard error) [8]. Mothers who have the highest DHA concentration in their breast milk typically live in coastal areas such as the Canadian Artic, Japan and the Dominican Republic, as these areas have high seafood intake [8]. These locales have DHA concentrations ranging from 0.6%–1.4% [8]. Jackson and Harris examined evidence toward obtaining a goal for DHA concentration in breast milk and suggested that 0.3% is a reasonable initial target as it provides some improvement over the typical Western diet DHA breast milk level of 0.2% [9]**.** Other studies have shown that it is possible to raise the DHA concentration in breast milk by maternal supplementation [10, 11]. Dunstan et al. demonstrated that women who were supplemented with fish oil starting in pregnancy had a significantly higher DHA level in their breast milk over the first 6 postpartum weeks compared to controls [11]. Another study randomized lactating mothers to receiving a placebo, 200 mg of DHA daily, or 400 mg of DHA daily and compared DHA concentrations in breast milk [10]. The authors found that not only was the DHA content of breast milk higher in the high dose DHA group but that after 6 weeks of maternal supplementation the infants in the high maternal dose DHA group also had the lowest n-6:n-3 ratio in their plasma [10]. This study concluded that maternal supplementation with DHA can help increase DHA concentration in breast milk and thus increase the amount of DHA that infants receive. Gestational age and stage of lactation has also been shown to have an effect on DHA content of breast milk. Breast milk from mothers who delivered preterm had a significantly higher initial DHA content compared to mothers who delivered at term [12]. DHA has also been shown to significantly decrease in breast milk over the first month of lactation in both term and preterm human milk samples [12]. While DHA decreases in breast milk over the first month of lactation, total fat, LA and ALA tend to increase [13]. Lastly, genetics can also contribute to the DHA content of breast milk. Fatty acid desaturase (FADS)1 and FADS2 code the enzymes ∆5-desaturase and ∆6-desaturase that are involved in the synthesis of ARA, EPA and DHA [14]. There is an association between maternal FADS genotype and the DHA content in breast milk with certain genotypes leading to a lower proportion of DHA in breast milk regardless of increased omega-3 intake [14, 15].

Donor breast milk is a significant component of nutrition for the premature infant and has been shown to have low amounts of DHA due to maternal diet as well as donor milk frequently coming from a later stage of lactation [16–19]. There is a large variance in DHA content in donor breast milk obtained through various donor milk banks across the United States [16]. DHA levels were found to vary from 0.07% to 0.2% in various studied United States milk banks [16]. A recent cross-sectional sample of five donor breast milk banks (Ohio, Michigan, Texas, Colorado, and California) found that the average DHA concentration in donor milk was 7.1 mg/100 ml which would provide preterm infants a DHA intake much lower than the fetal accretion rate of 42–67 mg/day in the third trimester [17, 20, 21]. Another study by Valentine et al. evaluating DHA concentration in donor breast milk in Ohio also found a low donor milk DHA concentration of ~0.1% [18]. In a subsequent study, Valentine et al. demonstrated that supplementing breast milk donors with 1 g of DHA daily increased the absolute milk concentration of DHA in donor breast milk to 4 times as high as the control group after 4 days of supplementation [19]. Based on an estimated intake of 150 ml/kg/day of donor milk in the preterm infant, the donor breast milk obtained from mothers receiving this DHA supplementation regimen would meet the expected intrauterine accretion levels for the preterm infant [19]. More recently, liquid human milk fortifier added to breast milk has been shown to increase the DHA content of breast milk [22]. Fortifier, with a DHA concentration of ~0.15%, can increase the level of DHA in the breast milk to 0.29%. This is considerably closer to the world wide mean DHA level of 0.32%, though still falls short of the intrauterine accretion rate. Per Lapillonne et al., milk containing a DHA concentration of ~0.8% given at 150 ml/kg/day provides 45 mg/kg/day of DHA which is very similar to the intrauterine accretion rate [13].

Breast and donor milk obtained from mothers eating a Western diet typically have low concentrations of DHA that is below the worldwide mean of 0.32%. By giving DHA supplements to mothers, it is possible to raise the DHA concentration of breast milk. Alternatively, for donor breast milk, where the milk is obtained from a later stage of lactation, using liquid human milk fortifier that contains DHA also raises DHA concentration level closer to the worldwide mean. This could potentially be vital for premature infants who typically are deficient in DHA.

### DHA and preterm infants

While DHA is important to the term infant, it is particularly critical to the premature infant who is delivered early in or prior to the third trimester. Overall, fetal DHA accretion is estimated to be 10 g, with the majority being acquired in the third trimester. [23]. The placenta aids with DHA accretion by allowing for preferential transfer of DHA over other fatty acids including LA and ALA [23]. As previously stated, the intrauterine accretion rate of DHA during the third trimester is approximately 42–67 mg/day or ~43 mg/kg/day [4, 5, 24]. This increase in DHA accretion in the third trimester coincides with brain and retina maturation [25]. DHA intake during pregnancy is not only important to ensure the fetus obtains adequate levels, but a systematic review by Kar et al. also showed that omega-3 fatty acids decrease the risk of delivery prior to 34 weeks by 58% [26]. Per Shireman et al., in a post-hoc analysis of their KUDOS study that showed a daily DHA intake of 600 mg led to decrease length of hospitalization for the infant; thus having mothers take daily DHA could lead to cost savings for the health care system [27].

Premature infants are typically deficient in DHA for several reasons including missing the in utero accumulation of DHA in the third trimester, their inability to convert precursor fatty acids to DHA in large amounts, and deficient postnatal DHA intake [16]. Infants delivered at the lowest gestational ages are at the highest risk for DHA deficiencies [28]. De Rooy et al. published a study that evaluated the levels of DHA in extremely premature infants receiving standard care [29]. This longitudinal study evaluated omega-6 and omega-3 intake from all sources in infants <28 weeks. The authors showed that the total amount of DHA that infants received at the end of 6 weeks represented only 36.6% of DHA that they would have received in utero [29]. Extremely low birth weight infants who are exposed to IV lipids for a prolonged time also have a significant decline in their DHA levels [30]. This decline in DHA was seen when evaluating RBC DHA levels and was more significant in ELBW infants who received intravenous lipids for greater than 28 days [30]. This is not surprising as Intralipid, the lipid emulsion commonly used in the United States, is virtually barren of DHA and was originally created for adults who have a greater capability of converting DHA from its precursor [31]. As breast milk typically does not have the quantity of DHA needed to make up for the deficiency in DHA in preterm infants, researchers have studied other ways to increase DHA levels in infants.

Collins et al. examined the effects of providing infants delivered at <33 weeks different doses of DHA [32]. Infants were randomized to receive 40 mg/kg/day, 80 mg/kg/day or 120 mg/kg/day of DHA orally for 28 days with the primary outcome being erythrocyte phospholipid DHA levels [32]. The authors found that supplementing infants with 120 mg/kg/day of DHA prevented the drop in DHA typically seen in premature infants at this age [32]. The benefit of this oral emulsion was that it allowed infants to receive a larger amount of DHA early on than what they would receive from breast milk, even from breast milk obtained from mothers taking supplementation. While this study discontinued supplements at 28 days, a study performed by Baack et al. randomized preterm infants to receive a DHA supplement or from the first week of life until term or discharge [33]. Infants in the DHA supplement group received 50 mg/day of DHA to match in utero DHA accretion rates. While DHA supplementation did significantly increase DHA levels in infants, the DHA levels at discharge in supplemented preterm infants remained significantly lower than term infant values [33]. Another recent study looking at delivering DHA via the buccal method found that this method did not alter DHA levels after 8 weeks of supplementation [34].

While term infants can achieve adequate DHA levels in breast milk through maternal supplementation, preterm infants struggle to overcome their DHA deficit. While directly supplementing preterm infants, as shown by both Collins et al. and Baack et al., does successfully increase their DHA level, the optimal amount of supplementation still needs to be determined [32, 33].

### DHA and cognitive/visual outcomes in preterm infants

DHA is one of the most abundant LCPUFAs in the brain and has an important role in brain development including roles in neurotransmission and neurogenesis [35–37]. It is also found in high levels in the visual system, especially in rod photoreceptors and M retinal ganglion cells [38]. From reviews of previous studies, the most consistent benefit from DHA supplementation was seen in earlier premature infants with lower birthweights, typically <1500 g, as these infants missed the majority of the third trimester with the highest fetal accretion rates of DHA. Therefore, a focus of DHA research has been to explore its role on cognitive and visual outcomes in the premature infant.

Previous studies have examined the association between DHA levels in the premature infant and cognitive outcomes. Tam et al. observed a cohort of 60 preterm infants between 24 and 32 weeks gestational age and noted an association between higher early red blood cell DHA levels and a decrease in both the incidence and severity of intraventricular hemorrhage (IVH) [39]. The authors showed that a 1% increase in early postnatal DHA was associated with a 4.3 fold decrease in the odds of intraventricular hemorrhage [39]. At 30–36 months corrected age (mean age of 33 months corrected), neurodevelopment outcome was able to be assessed in 45 of the 60 enrolled infants using the Bayley Scales of Infant Development, 3rd edition (BSID-3). The study showed an association with higher early DHA levels and improved developmental outcome at 30–36 months old that was not fully explained by the decreased risk of IVH [39]. Sabel et al. performed a longitudinal study on the development of premature infants at 3, 6, 10 and 18 months corrected age using the Bayley’s Scales of Infant Development, Second Edition (BSID-II) [40]. They found that in the initial 6 months, developmental scores were positively associated with breast milk DHA concentration [40]. Development was positively associated with DHA and negatively associated with n-6 fatty acids up to 18 months of age [40]. While these studies looked at the association between infant DHA level and cognitive outcome, there have been several studies evaluating the effects of DHA supplementation in premature infants on cognitive outcomes.

The DHA for the Improvement of Neurodevelopmental Outcome in preterm infants (DINO) trial was an Australian trial that randomized premature infants delivered at <33 weeks gestation to a high-DHA or standard-DHA diet until they reach 40 weeks corrected age [41]. Mothers of infants in the high-DHA group took 6 capsules daily, containing DHA-rich tuna oil and the mothers of infants in the standard-DHA diet received placebo soy capsules. When analyzing the breast milk of the two groups, the high-DHA group had milk with a mean DHA concentration of 0.85% versus 0.25% in the standard DHA group. At 18 months the Mental Development Index (MDI) of the BSID-II was performed. Overall, there was no statistically significant difference between the high DHA and the standard DHA group. Although the MDI in infants with birth weights <1250 g was higher, it did not reach statistical significance when adjusted for gestational age, sex, maternal education and birth order. When comparing only the girls in the high-DHA and standard-DHA groups, there was a statistically significant improvement in MDI scores in the high-DHA group [41]. The proportion of infants with severe developmental delay was also significantly less in the high-DHA group. Subsequently, the participants in this study were followed up at 7 years of age to assess their general intellectual ability using the Full Scale IQ of the Wechsler Abbreviated Scale of Intelligence (WASI) [42]. The researchers found no significant difference between the two groups in Full Scale, Verbal or Performance IQ [42].

While the DINO study supplemented mothers to achieve higher DHA levels in the breast milk, a randomized controlled trial by Henriksen et al. enrolled very low birth weight infants born at one of three hospitals in Norway and randomized them to receive a DHA/AA supplement or placebo for approximately 9 weeks after birth [43]. This supplement was directly added to their milk. The intervention group received supplemented human milk with a DHA concentration of 0.86% of total fatty acids, which is considered a high dose compared to the 0.3% level typical in preterm formula. At the corrected age of 6 months, the Ages and Stages Questionnaire was administered and the intervention group scored significantly higher on the problem solving section of the Ages and Stages Questionnaire compared to the control group [43]. There was also a trend toward an overall higher score in the intervention group that did not reach statistical significance (221 vs 215 points, *p*-value not given) [43]. In a study published by Almaas et al., the subjects were then followed up at 8 years of age, although no significant difference in cognitive functions was found between the two groups [44].

While the above studies demonstrated some early benefit with DHA supplementation, this benefit was not seen once the children reached school-age. A Cochrane review, updated in 2017, assessed whether giving premature infants formula milk supplemented with LCPUFA improved development, both cognitively and visually. The median concentration of DHA in formula in the included studies was 0.33% with a range of 0.15% to 1%. Based on their analysis, the authors concluded that premature infants did not obtain a benefit from receiving formula milk supplemented with LCPUFAs. Importantly, however, the review stated that outcomes should be evaluated based on a yet to be determined optimal dose [45].

Studies have also evaluated the effects of DHA on the development of visual function in the premature infant. The DIAMOND (DHA Intake and measurement of neural development) study, though done in term infants is an important study which evaluated how DHA supplementation of infant formula, administered in one of four concentrations (0%, 0.32%, 0.64%, or 0.96%), effected visual acuity in term infants at 12 months of age [46]. This study found that infants who received any of the formula supplemented with DHA had significantly better visual evoked potential (VEP) visual acuity at 12 months of age. There was no significant difference in VEP visual acuity between DHA concentrations, however, and the authors concluded that additional DHA above 0.32% had no additional benefit [46]. In another study, performed by Smithers et al., visual responses were assessed in premature infants at two months corrected age. The infants had been randomized into either a high-DHA group receiving 1% DHA or the standard-DHA group receiving 0.3%DHA. This study found that infants in the high-DHA group had significantly better visual acuity at 4 months corrected age compared to infants in the standard-DHA group [47]. Molloy et al. evaluated the effects of high-DHA versus standard-DHA on visual function at school age on a subgroup of children from the DINO trial described previously [48]. They assessed the participants’ visual function using the Freiburg Visual Acuity Test at 7 years old. The authors found that infants who received the human milk supplemented with high-DHA had no significant long-term benefit in their visual processing compared to infants who received standard-DHA [48].

Premature infants are deficit in DHA at birth and while short-term developmental benefits have been seen with DHA supplementation, no effects on long-term development have been shown. Because studies vary in the dosage, delivery, and length of DHA supplementation, it remains difficult to come to a decisive conclusion on DHA and cognitive outcomes. Until an optimal method for supplementing DHA to a preterm infant is found, it will continue to be a challenge to determine the true effect DHA supplementation has on the cognitive development of a premature infant.

### DHA and inflammation

Premature infants are at risk for inflammatory diseases such as bronchopulmonary dysplasia (BPD) and necrotizing enterocolitis (NEC), risks which may be mitigated by DHA’s anti-inflammatory properties. Atopic sensitization is also known to occur early in life and it is thought that omega-3 may help prevent this. As mentioned previously, without supplementation, DHA levels in preterm infants drop following birth [31]. Martin et al. showed an association between DHA levels in the first four weeks of life and the development of chronic lung disease (CLD) in premature infants. Lower DHA levels in these infants were significantly associated with the development of CLD. This study showed that a 1% decrease in the total fatty acid mass of DHA in the blood gave a 2.5 fold increase in the odds of developing CLD [31]. Similarly for late-onset sepsis, the authors found an association between decreased DHA concentration and an increased risk for developing late-onset sepsis. Lower AA and higher LA levels were also significantly associated with the development of late-onset sepsis in this cohort of premature infants. The authors did not find an association between mean DHA levels and IVH, NEC or ROP [31]. In addition to DHA levels, the authors also found that increased ratios of LA:DHA, AA:DHA, and n-6: n-3 were associated with CLD. A similar study was completed in Tunisia evaluating plasma DHA levels where improved control of the n-6:n-3 ratio may help prevent certain neonatal morbidities, such as RDS, sepsis and IVH in the premature infant [49]. Because infants who are born extremely premature often require TPN, a study in Greece examined the effects of various lipid emulsions on certain cytokine levels known to be proinflammatory: tumor necrosis factor (TNF)-α, interleukin (IL)-6, and IL-8. Infants who received a lipid emulsion which contained omega-3’s had significantly lower IL-6 and IL-8 levels compared to infants receiving a typical soybean oil-based lipid emulsion [50]. Prior studies have shown an association with these cytokines and BPD in premature infants and subsequent studies have evaluated the effects of DHA on BPD in premature infants [51–53]. Another study showed that maternal fish oil supplementation of 1.5 g/day of n-3 long-chain PUFA can continue to have an effect on a child’s immune system up to at least two years after the supplement was given [54].

In a follow-up to the DINO trial that initially evaluated cognitive outcomes with DHA supplementation in premature infants, the authors evaluated the long-term respiratory and allergy outcomes in these infants. The infants were evaluated at 18 months for history of BPD as well as parental reports of atopic conditions such as hay fever, asthma, eczema or food allergies [52]. Of the 657 infants originally enrolled in the study 614 infants completed the 18-month follow-up, though the allergy data was incomplete, as some families did not complete these questionnaires. The authors found a statistically significant reduction in parental reported hay fever at either 12 months or 18 months in the high –DHA group compared to the standard DHA group, but no reported reductions in food allergies, eczema or asthma. The risk of developing BPD was not significantly different between the two groups but there was a significant reduction of oxygen requirement at 36 weeks in both male infants and infants with birth weights <1250 g. Of note, these findings should be interpreted with caution as the study was not powered to detect these differences [52].

Based on these previous studies suggesting that DHA may reduce the risk of BPD, the N-3 Fatty Acids for Improvement in Respiratory Outcomes (N3RO) study was developed [55]. This was a large randomized, blinded controlled trial conducted in Australia, New Zealand and Singapore that was designed to assess the effect of DHA given to premature infants delivered prior to 29 weeks on the incidence of BPD [55]. This study randomized 1273 infants to receiving either 60 mg/kg/day DHA enteral emulsion or to receive no additional DHA. There was no statistically significant difference in the development of BPD between the groups and though not significant, the DHA group actually had a higher percentage of infants who developed physiological BPD (49.1% vs 43.9%, *p*-value 0.07) [55]. The authors concluded that supplementing premature infants with DHA enterally did not reduce the incidence of BPD and actually may have increased the risk [55].

## Conclusion

DHA is an important LCPUFA that has roles in several physiological processes including brain and visual development as well as the inflammatory process. Mothers in the United States typically consume a Western diet that is low in omega-3 fatty acids which can also lead to breast milk that has a low concentration of DHA. Premature infants, especially those born before 30 weeks, have been shown to have a deficit in DHA shortly after delivery for several reasons including foregoing third trimester DHA accretion, receiving enteral breast milk or donor milk feeds that are typically low in DHA, and having an increased risk of requiring prolonged TPN. Despite numerous studies evaluating the effects of DHA on development in the premature infant, there are still conflicting results on whether DHA actually improves the cognitive outcomes in premature infants. While short-term benefits have been seen in several studies, long-term benefits are not consistent. Future studies continue to be needed to assess the optimal DHA dosage, method of delivery (maternal supplementation, direct supplementation, IV lipid emulsions, etc.), and length of supplementation to optimize DHA intake in very premature infants.

## Abbreviations

ALA: α-linolenic acid
BPD: Bronchopulmonary dysplasia
BSID: Bayley Scales of Infant Development
CLD: Chronic lung disease
DHA: Doxosahexaenoic acid
FADS: Fatty acid desaturase
IVH: Intraventricular hemorrhage
LA: Linoleic acid
LCPUFA: Long-chain polyunsaturated fatty acids
MDI: Mental Development Index
NEC: Necrotizing enterocolitis
ROP: Retinopathy of prematurity
TNF: Tumor necrosis factor
VEP: Visual evoked potential
WASI: Wechsler Abbreviated Scale of Intelligence
